# Sulfated polysaccharides interact with fibroblast growth factors and protect from denaturation

**DOI:** 10.1002/2211-5463.12696

**Published:** 2019-07-16

**Authors:** Changye Sun, Mengxin Liu, Panwen Sun, Mingming Yang, Edwin A. Yates, Zhikun Guo, David G. Fernig

**Affiliations:** ^1^ Henan Key Laboratory of Medical Tissue Regeneration Xinxiang Medical University China; ^2^ Department of Cardiology School of Medicine Affiliated Zhongda Hospital Southeast University Nanjing China; ^3^ Department of Biochemistry Institute of Integrative Biology University of Liverpool UK

**Keywords:** differential scanning fluorimetry, fibroblast growth factor, protein stability, sulfated polysaccharide

## Abstract

Fibroblast growth factors (FGFs) regulate embryonic development and homeostasis, including tissue and organ repair and specific aspects of metabolism. The basic FGF and acidic FGF, now known as FGF2 and FGF1, are widely used protein drugs for tissue repair. However, they are susceptible to denaturation at ambient temperatures and during long‐time storage, which will reduce their biological activity. The interaction of FGFs with the sulfated domains of heparan sulfate and heparin is essential for their cellular signaling and stability. Therefore, we analyzed the interactions of FGF1 and FGF2 with four sulfated polysaccharides: heparin, dextran sulfate (DXS), λ‐carrageenan, and chondroitin sulfate. The results of thermal stability and cell proliferation assays demonstrate that heparin, DXS, and λ‐carrageenan bound to both FGFs and protected them from denaturation. Our results suggest heparin, DXS, and λ‐carrageenan are potential formulation materials that bind and stabilize FGFs, and which may also potentiate their activity and control their delivery.

**Abbreviations**
CCK‐8: Cell Counting Kit‐8
CS: chondroitin sulfate
DMEM: Dulbecco's modified Eagle's medium
DSF: differential scanning fluorimetry
DXS: dextran sulfate
FGF: fibroblast growth factors
FGFR: FGF receptors
GalNAc: *N*‐acetyl‐d‐galactosamine
HS: heparan sulfate
Tm: melting temperature

Fibroblast growth factors (FGF) regulate embryonic development and homeostasis, including the repair of tissues and organs and specific aspects of metabolism 1, 2. The basic FGF and acidic FGF, now known as FGF2 and FGF1, respectively, are the most studied FGFs and have been developed as therapeutics 1. Along with the other paracrine FGFs, they bind to heparan sulfate (HS) chains and interact with FGF receptors (FGFR) (Fig. 1A). Activation of the FGFR by the binding of FGF ligand and HS co‐receptor induces the activation of the FGFR kinase 3, 4, 5. This phosphorylates its targets, leading to the activation of many intracellular signaling pathways, for example, RAS‐RAF‐MAPK and PI3K‐AKT, which regulate cell fate and specific cell activities 2, 6, 7, 8.

**Figure 1:**
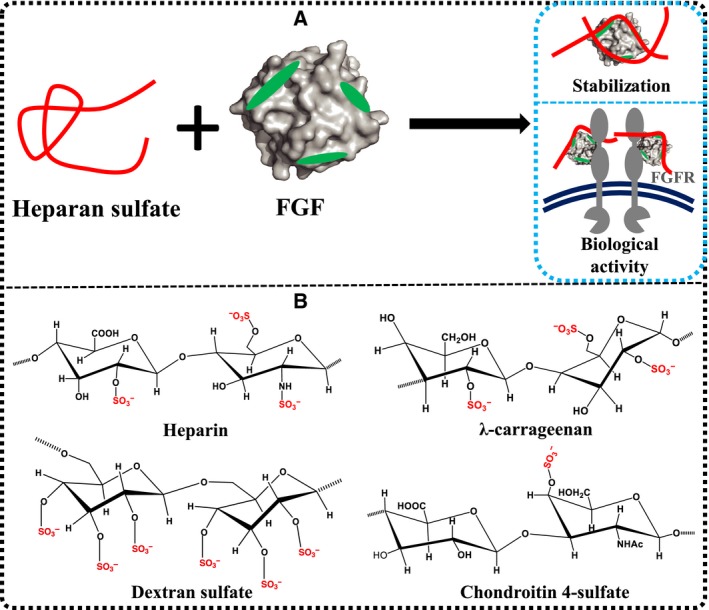
Structural interaction of HS, FGF, and FGFR and the distribution of sulfate groups in heparin, λ‐carrageenan, DXS, and CS. (A) FGF1 and FGF2 contain three HS binding sites in green which can interact with HS 19, 32. This interaction can stabilize the FGF protein and form a stable complex with FGFR competent to generate signals in the cytoplasm. (B) Structures of the disaccharide units of heparin, λ‐carrageenan, DXS, and CS with sulfate groups in red. For heparin, this is the structure of the most common disaccharide unit, and 25% of disaccharides have fewer sulfate groups. HS has the same disaccharide structure as heparin, but is less sulfated 33, 34.

Since FGF1 is the universal FGF ligand and so activates all FGFRs, and FGF2 activates ‘c’ isoform receptors, they possess multiple activities in development and in homeostasis, for example, mitogenic, neurotrophic and angiogenic activities 9, 10. Consequently, FGF1 and FGF2 have been developed as therapeutics for tissue repair. For example, FGF1 and FGF2 are used to accelerate wound healing in skin 1, 11. FGF1 and FGF2 (natural or *Escherichia coli*‐derived) have potent activities on many cell types, but their activity, especially that of FGF1, reduces over time, even at body temperature 12, 13, 14, 15, 16. Temperature‐depended denaturation and degradation by enzymes are likely to contribute to limiting the efficacy of these FGFs 17, 18, 19, 20, 21.

As well as enabling the formation of an active signaling complex with the FGFR (Fig. 1A), the interaction of the paracrine FGFs with heparin (Fig. 1B) or the related physiologically relevant polysaccharide, HS, increases their stability, including resistance to heat‐induced denaturation and proteolysis 22. Binding to HS also regulates their diffusion from source to target cell in tissues, and so their gradients and bioavailability 23, 24, 25. A large body of work demonstrates that the interaction of FGFs with HS/heparin is mainly mediated by the sulfate groups on the polysaccharides 26. However, heparin is expensive and it also has strong anti‐coagulation activity, and so is not suitable as an activity enhancing component of a FGF therapeutic formulation 27, 28. Another common commercial animal‐derived sulfated polysaccharide is chondroitin sulfate (CS), which has a *N*‐acetyl‐d‐galactosamine (GalNAc) amino sugar, beta 1–3 linkages and is less sulfated (Fig. 1B), and is considered to only bind weakly to just a subset of FGFs 19, 29. Two highly sulfated polysaccharides are dextran sulfate (DXS) (six sulfate groups per disaccharide unit) and λ‐carrageenan (three sulfate groups per disaccharide unit) (Fig. 1B), which like CS are cheap and, due to their higher sulfation, may bind FGFs and so be potential additives for FGF protein formulations 30, 31. In the present work, we aim to determine the stabilization effects of DXS and λ‐carrageenan on FGF1 and FGF2 and the ability of these polysaccharide chains to protect the biological activity of these FGFs.

## Materials and methods

### Materials

**Figure 2:**
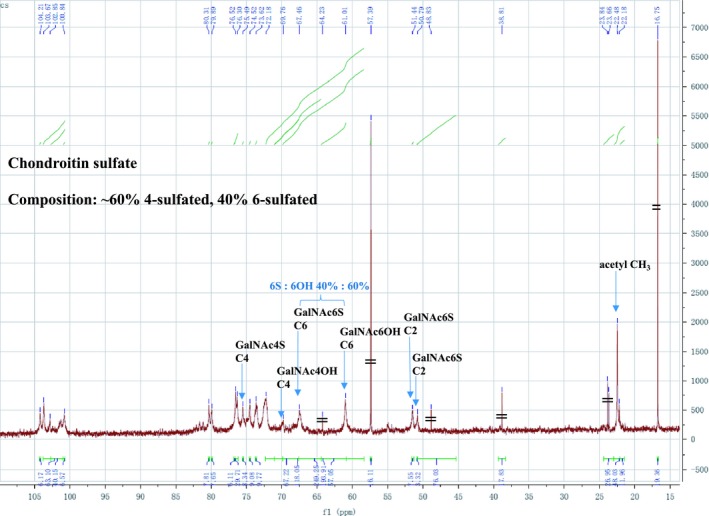
Analysis of sulfation degree of CS. The C^13^ spectrum of the CS was analyzed with nuclear magnetic resonance (Acend 400 NMR, Bruker BioSpin, Bruker Scientific Technology Co., Ltd., Shanghai, China) by scanning for 20 h. The GalNAc signal comprises 40% 6‐O‐sulfate and 60% 6‐OH groups, which indicates that the polysaccharide is a mixture of chondroitin 4‐sulfate (60%) and chondroitin 6‐sulfate (40%).

The polysaccharides were purchased from Sigma (St. Louis, MO, USA, heparin, 18 kDa average molecular mass; λ‐carrageenan) and Solarbio (Beijing, China, DXS, 500 kDa average molecular mass and CS, 5–20 kDa). Since CS is often a mixture of species, this was analyzed by NMR 35 (Fig. 2). The data indicate that it is 60% chondroitin 4‐sulfate, 40% chondroitin 6‐sulfate, with other polysaccharides at too low a level to be reliably detected. *E. coli* strain (BL21) for protein expression was bought from Transgen Biotech (Beijing, China). Heparin affinity gel (Bio‐Rad, Hercules, CA, USA), nickel affinity gel, and SP Sepharose Fast Flow (GE Healthcare Life Sciences, Uppsala, Sweden) were used for FGF protein purification. SYPRO Orange dye and optical 96‐well plates were from Thermo Scientific (Eugene, OR, USA). The 293T cell line was from the stem cell bank of the Chinese Academy of Sciences (Shanghai, China). For 293T cell culture, the following materials were used: Dulbecco's modified Eagle's medium (DMEM, containing 4500 mg·L^−1^ glucose and 4 mm l‐Glutamine; HyClone Laboratories, Logan, UT, USA; GE Healthcare Life Sciences), FBS (PAN Seratech, Aidenbach, Germany), PBS, trypsin, and penicillin–streptomycin (all Solarbio); cell culture dishes and 96‐well plates were from Corning (Oneonta, NY, USA) and the Cell Counting Kit‐8 (CCK‐8) for detecting cell proliferation was bought from Dojindo (Shanghai, China).

### FGF protein preparation

FGF1 and FGF2 proteins with a hexahistidine tag were expressed and purified as described 14, 36. Briefly, FGF1 and FGF2 were expressed in BL21 cells at 18 °C for 18 h. The bacteria containing FGF protein were harvested and stored at −80 °C. The bacterial pellets were resuspended in lysis buffer (50 mm Tris/Cl and 0.6 m NaCl, pH 7.4), and the cells were disrupted by sonication on ice. Cell debris and insoluble proteins were pelleted by centrifugation at 4 °C, 30 000 ***g*** for 30 min. The supernatant containing FGF protein was purified by heparin affinity‐gel chromatography. The eluate was further purified with nickel affinity chromatography and SP Sepharose Fast Flow chromatography to remove any remaining impurities, and the purified FGF protein was stored at −80 °C. Purification was confirmed by SDS/PAGE.

### Protein thermal stability assay

Heparin powder (500 mg) was dissolved in 50 mL PBS to prepare a 10 mg·mL^−1^ heparin solution for differential scanning fluorimetry (DSF) assays. DXS (10 mg·mL^−1^), λ‐carrageenan (10 mg·mL^−1^), and CS (10 mg·mL^−1^) were prepared in the same way. Each polysaccharide was diluted with PBS to prepare a range of concentrations.

Differential scanning fluorimetry was used to detect the thermal stability of FGFs, as described 26. The purified FGFs (5 μm), polysaccharide ligands (8 μL of a particular concentrations), SYPRO Orange dye (100×, 4 μL), and PBS (added to make up total volume to 40 μL; 137 mm NaCl, 2.7 mm KCl, 10 mm Na_2_HPO_4_, and 1.8 mm KH_2_PO_4_, pH 7.4) were mixed, and 10 μL added to each of three wells in an optical 96‐well plate 26, 37. The plate was then covered with Optical Adhesive Film to prevent evaporation. The melting curve data were acquired with a QuantStudio™ 7 Flex Real‐Time PCR machine (Applied Biosystems Life Technologies, Woodlands, Singapore). The running method was designed to raise the temperature from 32 °C to 81 °C (for FGF1) and to 92 °C (for FGF2) at a heating rate of 0.05 °C·s^−1^. The raw data were exported for further analysis of melting temperature (Tm) after measurement.

### DSF data analysis

The DSF data were analyzed with SimpleDSFviewer to obtain melting curves, first derivative curves and so the Tm 38. The Tm was calculated by the half denaturation method 38. The melting curve and first derivative curve were plotted in Excel, and the averaged Tm with standard deviation was plotted in matlab 2015a (The MathWorks, Natick, MA, USA).

### Cell culture and cell growth assay

293T cells were cultured in DMEM containing 10% (v/v) FBS and 1% (v/v) penicillin–streptomycin. For cell growth assays, 293T cells were dispensed into 96‐well plates at 2000 cells/well. After 24‐h incubation, the cells were washed twice with PBS and cultured in DMEM containing 0.5% (v/v) FBS for another 24 h. FGF1, FGF2, and polysaccharides were added to the wells for 36 h, as described in the figure legends, after which CCK‐8 (10 μL) was added to each for 2 h. The absorbance at 450 nm was then measured using a microplate reader (SpextraMax i3; Molecular Devices, San Jose, CA, USA).

To detect the thermal protection effect of polysaccharides, FGF1 and FGF2 were diluted to 400 ng·mL^−1^ with DMEM with or without polysaccharide (1 μg·mL^−1^). The samples were incubated at 37 °C for 1 or 2 days and then added to cells in 96‐well plates. The cell growth assay protocol was then followed; 293T cells were seeded into 96‐well plates and starved with DMEM containing 0.5% (v/v) FBS (90 μL) for 24 h. Then, 30 μL of the pre‐incubated samples of the FGFs was added to obtain a final concentration of 100 ng·mL^−1^ FGF with or without polysaccharide in each well.

All of the acquired absorbance values (450 nm) for CCK‐8 assay were normalized to the absorbance of the DMEM control group, and the results were analyzed and presented in matlab 2015a. Statistical analysis was performed with originpro 2019 software (OriginLab Corporation, Northampton, MA, USA). Multiple comparisons were analyzed by one‐way analysis of variance with the Tukey test. *P *<* *0.05 was considered significantly different.

## Results and Discussion

### Determination of interactions of FGFs with sulfated polysaccharide chains

**Figure 3:**
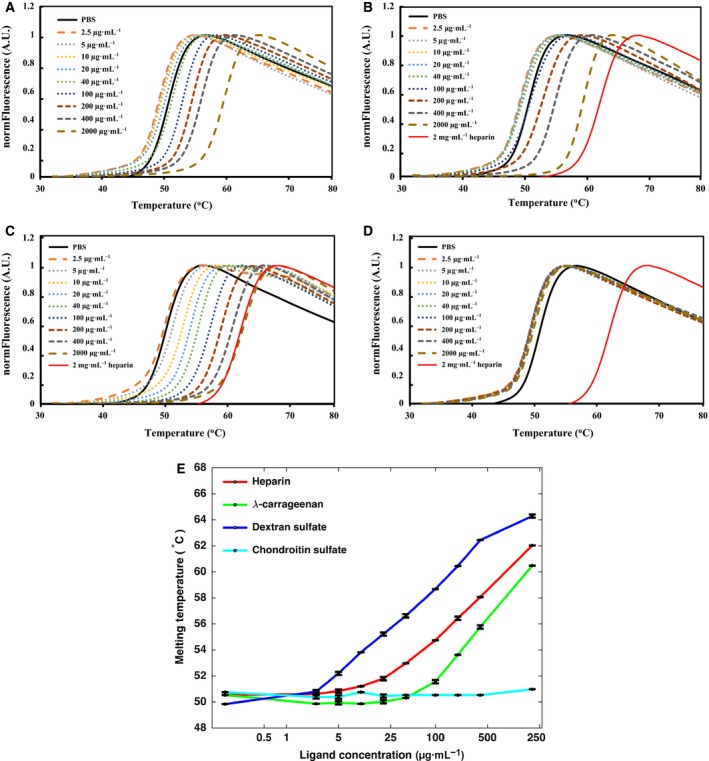
Stabilization effect of polysaccharide ligands (heparin, λ‐carrageenan, DXS, and CS) on FGF1. DSF of FGF1 (5 μm) in the presence of varying concentrations of polysaccharide ligands (0–2000 μg·mL^−1^) in PBS (pH 7.4). Melting curve profiles of FGF1 with a range of heparin (A), λ‐carrageenan (B), DXS (C), and CS (D). (E) Melting temperatures of FGF1 stabilized with polysaccharide ligands, mean of triplicates ± SD.

**Figure 4:**
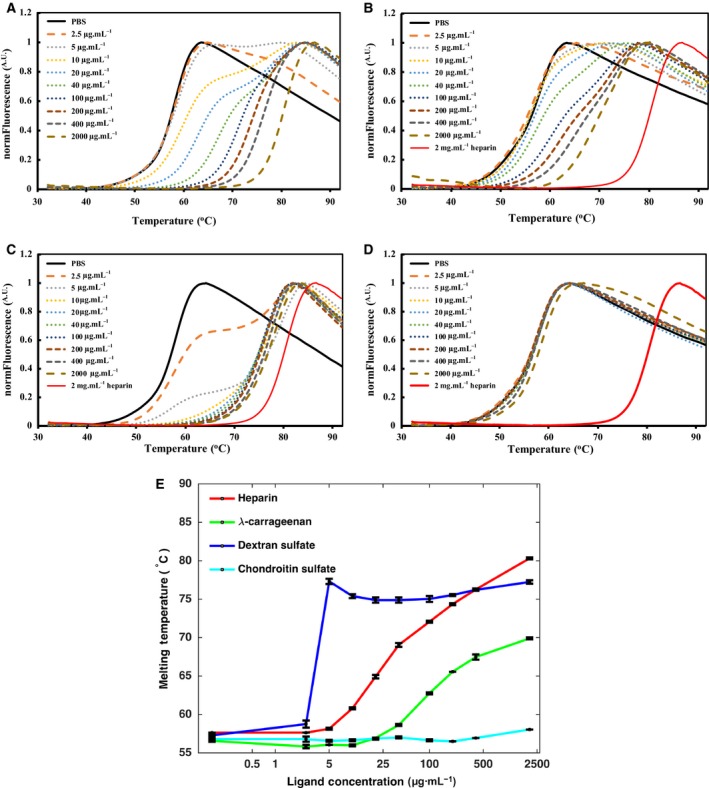
Stabilization effect of polysaccharide ligands (heparin, λ‐carrageenan, DXS, and CS) on FGF2. DSF of FGF2 (5 μm) in the presence of varying concentrations of polysaccharide ligands (0–2000 μg·mL^−1^) in PBS (pH 7.4). Melting curve profiles of FGF2 with a range of heparin (A), λ‐carrageenan (B), DXS (C), and CS (D). (E) Melting temperatures of FGF2 stabilized with polysaccharide ligands are the mean of triplicates ± SD.

FGF1 and FGF2 were purified by heparin affinity and nickel affinity chromatography. The melting curves of FGF1 and FGF2 (Figs 3 and 4) indicated a Tm of FGF1 of 49.5 °C and of 56.5 °C for FGF2. These data are entirely consistent with those previously published 15, 26, which demonstrates that the purified FGF1 and FGF2 were correctly folded.

In the presence of heparin, the melting curve of FGF1 shifted to the right, indicating a stabilization of the protein through its interactions with the polysaccharide (Fig. 3A). This stabilization effect was first apparent at 20 μg·mL^−1^ heparin (Tm 51.8 °C, Fig. 3A) and reached a maximum at 2000 μg·mL^−1^ heparin (Tm 62.0 °C, Fig. 3A). Since λ‐carrageenan and DXS are both highly sulfated polysaccharides, their ability to interact with FGF1 was tested. The DSF results indicate that FGF1 binds strongly to both λ‐carrageenan and DXS, since it is stabilized by both polysaccharides (Fig. 3B,C,E). Moreover, the stabilization effect is concentration‐dependent. The stabilization effect of λ‐carrageenan was first observed at 100 μg·mL^−1^ (Tm 51.5 °C, Fig. 3B), and the Tm reached the maximum (Tm 60.5 °C, Fig. 3B) at 2000 μg·mL^−1^. Thus, the stabilization effect of λ‐carrageenan is significantly weaker than that of heparin (*P *<* *0.05). DXS possesses the highest sulfation level, and its stabilization effect was first apparent at 2.5 μg·mL^−1^ (Tm 50.8 °C, Fig. 3C). The melting curve then shifted to the right as the concentration of DXS was increased, and the Tm reached 64.3 °C at 2000 μg·mL^−1^ DXS (Fig. 3C). DXS was, therefore, more effective at stabilizing FGF1 than heparin. In contrast, CS did not have a detectable stabilization effect on FGF1, which may reflect its lower sulfation or different backbone (Fig. 3D) or both.

The DSF results indicate that FGF2 is a more stable protein than FGF1 (Figs 3 and 4), which is consistent with the previous studies 15, 20. The Tm of FGF2 increased at 5 μg·mL^−1^ heparin (Tm 58.1 °C, Fig. 4A) and was highest at 2000 μg·mL^−1^ heparin (Tm 80.3 °C, Fig. 4A,E). Similarly to heparin, λ‐carrageenan also increased the Tm of FGF2 from 58.6 °C at 40 μg·mL^−1^ to 69.9 °C at 2000 μg·mL^−1^ (Fig. 4B,E). When FGF2 was stabilized by DXS, the melting curves showed two FGF2 melting states (not stabilized FGF2 and polysaccharide‐stabilized FGF2) (Fig. 4C,E). These indicate that over the timescale of the temperature change DXS does not dissociate from FGF2, so there is no averaging of the Tm. This effect is apparent, but much weaker in the melting curves of FGF2 in the presence of heparin and λ‐carrageenan. The stabilization of FGF2 by DXS was 77.2 °C (Fig. 4C). As with FGF1, CS did not have a detectable stabilization effect on FGF2 (Fig. 4D).

### Stimulation of 293T cell growth by FGFs or polysaccharides

**Figure 5:**
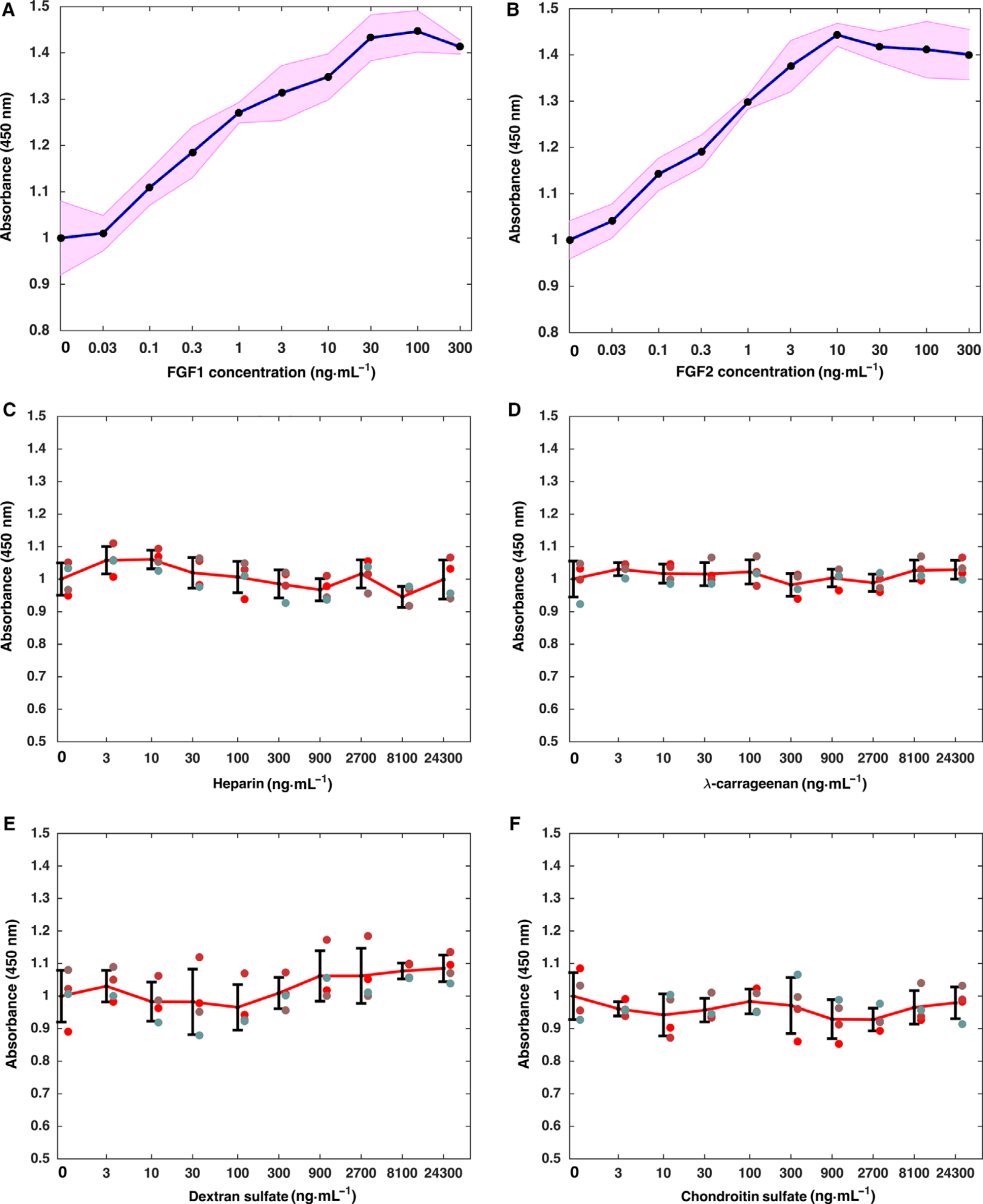
Biological activity of FGFs and polysaccharides on 293T cells. 293T cells were treated with different concentration of FGFs (0–300 ng·mL^−1^) and polysaccharides (0–30 000 ng·mL^−1^) for 36 h, and the cell proliferation was measured with CCK‐8. (A) FGF1; (B) FGF2; (C) heparin; (D) λ‐carrageenan; (E) DXS; (F) CS. Results are the mean of quadruplicates after normalization, with the ±SD region shaded (A, B) or ±SD bars (C–F).

293T cells were used to measure the biological activities of FGF1 and FGF2 and of the polysaccharides. When 0.03 ng·mL^−1^ FGF1 and FGF2 were added to culture medium, there was no change in cell growth (Fig. 5A,B). However, cell growth was observed to increase at 0.1 ng·mL^−1^ FGF1 or FGF2 (Fig. 5A,B). As the concentration of the growth factors was further increased, so did cell growth, until a maximum was reached at 30 ng·mL^−1^ FGF1 and 10 ng·mL^−1^ FGF2 (Fig. 5A,B). These results are consistent with the NIH 3T3 cell growth assays of the same growth factors 39.

In the presence of heparin, λ‐carrageenan, DXS, and CS alone, no effect on the growth of 293T cells was observed (Fig. 5C–F). Thus, these four polysaccharides by themselves have no growth‐stimulatory or toxic effect on the cells.

### Protection of FGFs’ biological activities by polysaccharides

**Figure 6:**
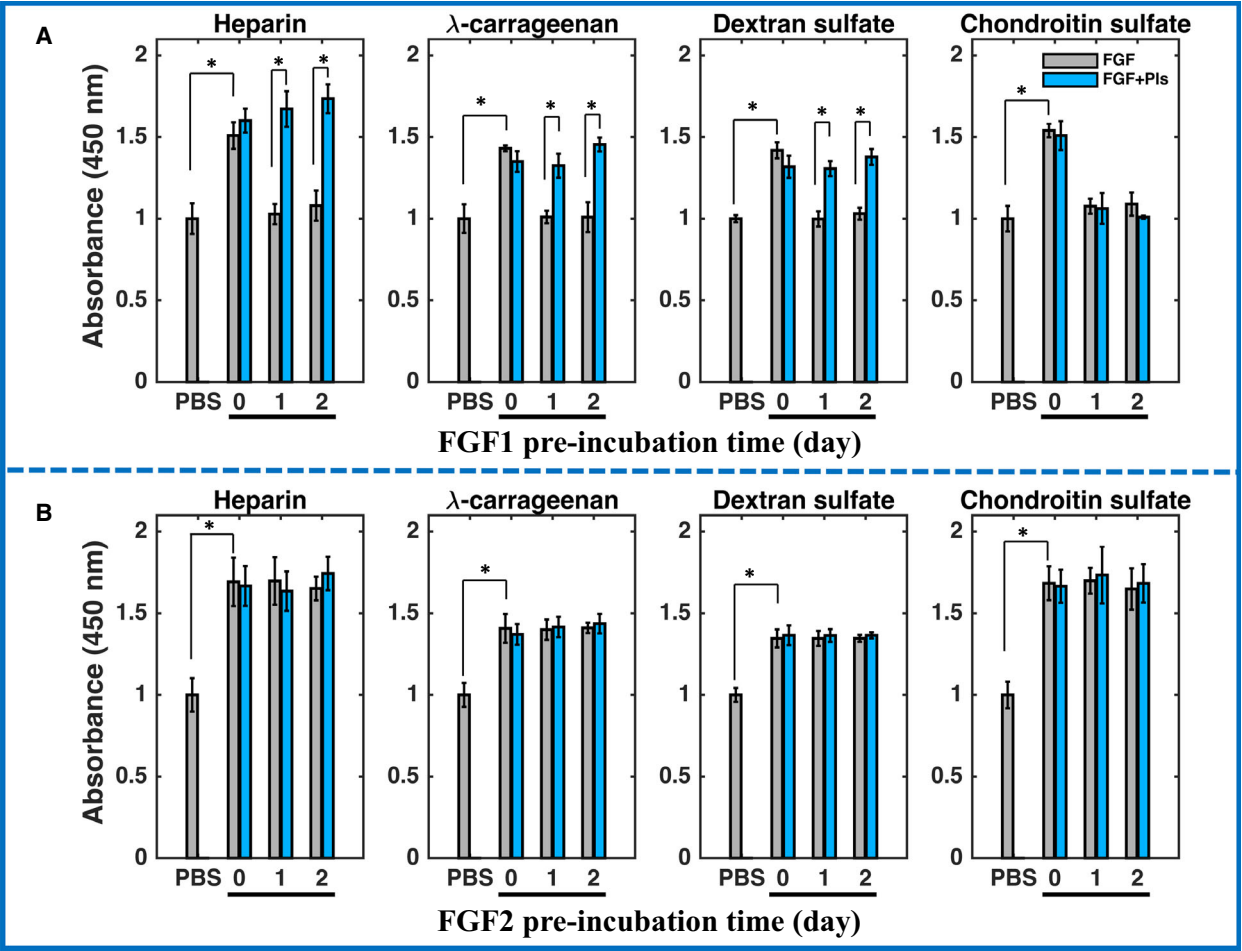
Protection of biological activities of FGF1 and FGF2 by polysaccharides. FGF1 (A) and FGF2 (B) were pre‐incubated with and without polysaccharide ligands (heparin, λ‐carrageenan, DXS, and CS) for 0 day, 1 day, and 2 days at 37 °C. Their biological activities were measured in 293T cells by the CCK‐8 assay, and data were normalized to the PBS control. FGF+Pls: FGF with the respective polysaccharide. Results are the mean of quadruplicates after normalization ±SD and statistical analysis performed with the Tukey test. *P <* *0.05.

To detect whether the selected polysaccharides can protect the FGFs’ stability and biological activity, FGF1 and FGF2 (400 ng·mL^−1^ in DMEM) were pre‐incubated at 37 °C for 1 day and 2 days to mimic the effect of long‐term exposure to body temperature. A final concentration of FGF1 and FGF2 of 100 ng·mL^−1^ was chosen, since this elicited a maximal response after 2 days (Fig. 5A,B). The results demonstrate that FGF1 directly taken from the freezer (0 day pre‐incubation) possessed significant biological activity on 293T cells comparing to the PBS control (Fig. 6A). After 1 day pre‐incubation at 37 °C, the biological activity of FGF1 was no longer apparent, indicating the protein had likely denatured over this time.

However, when heparin, λ‐carrageenan, and DXS were added, FGF1 was stabilized and even after the 2 days pre‐incubation the FGF1 had the same biological activity as FGF1 taken directly taken from the freezer (Fig. 6A). These results suggest that heparin, λ‐carrageenan, and DXS, which bind FGF1 and increase its Tm (Fig. 3E) protect its biological activity. Addition of CS did not show a detectable effect on the protection of FGF1's biological activity, which is consistent with this polysaccharide not increasing the Tm of FGF1 (Fig. 3E). FGF2 is intrinsically a more stable protein than FGF1, as shown in Figs 3 and 4. Similarly to FGF1, FGF2 shows a strong stimulation of cell growth on 293T cells (Fig. 6B). Moreover, after 2 days pre‐incubation at 37 °C the activity of FGF2 on 293T cells remained the same, which suggests addition of the four polysaccharides is not required for the protection of FGF2's biological activity.

## Conclusion

FGF1 and FGF2 have very different thermal stabilities, as illustrated by their Tm measured by DSF, 49.5 °C for FGF1 and 56.5 °C for FGF2. Indeed, FGF1 has been considered to be in a molten globule state at 37 °C 40. The thermal stability of FGF1 and FGF2 is substantially enhanced by binding to heparin (Figs 3E and 4E 26). It is interesting that the stabilization of FGF1 by heparin has been observed in cultured cells, which have pericellular HS capable of binding and stabilizing the growth factor 41, 42. This is likely due to the lower affinity of FGF1 for HS, compared with FGF2 41, such that a substantial amount of the protein will partition into the bulk culture medium rather than onto pericellular matrix HS. Heparin has a strong anticoagulant activity and is relatively expensive. However, we show here that other highly sulfated polysaccharides, DXS and λ‐carrageenan, can bind to FGF1 and FGF2 and increase their thermal stability. Moreover, these are able to stabilize FGF1 and ensure that it retains its activity for longer times. Thus, biomaterials containing λ‐carrageenan or DXS would be suitable for FGF drug delivery and would enhance the activity of FGF1, allowing lower doses to be used.

## Conflict of interest

The authors declare no conflict of interest.

## Author contributions

CS, EAY, ZG, and DGF conceived and designed the project. CS, ML, PS, EAY, and MY performed the experiments and analyzed the data. CS prepared figures and wrote the paper. DGF reviewed and revised the drafts of the paper.

## References

[1] Nunes QM , Li Y , Sun C , Kinnunen TK and Fernig DG (2016) Fibroblast growth factors as tissue repair and regeneration therapeutics. PeerJ 4, e1535. 26793421 10.7717/peerj.1535 PMC4715458

[2] Turner N and Grose R (2010) Fibroblast growth factor signalling: from development to cancer. Nat Rev Cancer 10, 116–129. 20094046 10.1038/nrc2780

[3] Delehedde M , Lyon M , Gallagher JT , Rudland PS and Fernig DG (2002) Fibroblast growth factor‐2 binds to small heparin‐derived oligosaccharides and stimulates a sustained phosphorylation of p42/44 mitogen‐activated protein kinase and proliferation of rat mammary fibroblasts. Biochem J 366, 235–244. 12000311 10.1042/BJ20011718 PMC1222755

[4] McKeehan WL , Wang F and Kan M (1998) The heparan sulfate fibroblast growth factor family: diversity of structure and function. Prog Nucleic Acid Res Mol Biol 59, 135–176. 9427842 10.1016/s0079-6603(08)61031-4

[5] Ornitz DM (2000) FGFs, heparan sulfate and FGFRs: complex interactions essential for development. BioEssays 22, 108–112. 10655030 10.1002/(SICI)1521-1878(200002)22:2<108::AID-BIES2>3.0.CO;2-M

[6] Goetz R and Mohammadi M (2013) Exploring mechanisms of FGF signalling through the lens of structural biology. Nat Rev Mol Cell Biol 14, 166–180. 23403721 10.1038/nrm3528 PMC3695728

[7] Delehedde M , Seve M , Sergeant N , Wartelle I , Lyon M , Rudland PS and Fernig DG (2000) Fibroblast growth factor‐2 stimulation of p42/44(MAPK) phosphorylation and I kappa B degradation is regulated by heparan sulfate/heparin in rat mammary fibroblasts. J Biol Chem 275, 33905–33910. 10944532 10.1074/jbc.M005949200

[8] Zhu HY , Duchesne L , Rudland PS and Fernig DG (2010) The heparan sulfate co‐receptor and the concentration of fibroblast growth factor‐2 independently elicit different signalling patterns from the fibroblast growth factor receptor. Cell Commun Signal 8, 14. 20576134 10.1186/1478-811X-8-14 PMC2912315

[9] Beenken A and Mohammadi M (2009) The FGF family: biology, pathophysiology and therapy. Nat Rev Drug Discovery 8, 235–253. 19247306 10.1038/nrd2792 PMC3684054

[10] Zhang XQ , Ibrahimi OA , Olsen SK , Umemori H , Mohammadi M and Ornitz DM (2006) Receptor specificity of the fibroblast growth factor family – the complete mammalian FGF family. J Biol Chem 281, 15694–15700. 16597617 10.1074/jbc.M601252200 PMC2080618

[11] El Agha E , Kosanovic D , Schermuly RT and Bellusci S (2016) Role of fibroblast growth factors in organ regeneration and repair. Semin Cell Dev Biol 53, 76–84. 26459973 10.1016/j.semcdb.2015.10.009

[12] Knoerzer W , Binder HP , Schneider K , Gruss P , Mccarthy JEG and Risau W (1989) Expression of synthetic genes encoding bovine and human basic fibroblast growth‐factors (bFGFs) in *Escherichia coli* . Gene 75, 21–30. 2470650 10.1016/0378-1119(89)90379-x

[13] Ke YQ , Fernig DG , Smith JA , Wilkinson MC , Anandappa SY , Rudland PS and Barraclough R (1990) High level production of human acidic fibroblast growth factor in *Escherichia coli* cells inhibition of DNA synthesis in Rat mammary fibroblasts at high concentrations of growth factor. Biochem Biophys Res Comm 171, 963–971. 1699532 10.1016/0006-291x(90)90778-l

[14] Ke YQ , Wilkinson MC , Fernig DG , Smith JA , Rudland PS and Barraclough R (1992) A rapid procedure for production of human basic fibroblast growth factor in *Escherichia coli* cells. Biochem Biophys Acta 1131, 307–310. 1627646 10.1016/0167-4781(92)90029-y

[15] Buchtova M , Chaloupkova R , Zakrzewska M , Vesela I , Cela P , Barathova J , Gudernova I , Zajickova R , Trantirek L , Martin J *et al* (2015) Instability restricts signaling of multiple fibroblast growth factors. Cell Mol Life Sci 72, 2445–2459. 25854632 10.1007/s00018-015-1856-8 PMC11113989

[16] Cirkovas A and Sereikaite J (2011) Different effects of (L)‐arginine on the heat‐induced unfolding and aggregation of proteins. Biologicals 39, 181–188. 21550265 10.1016/j.biologicals.2011.04.003

[17] Turnbull JE , Fernig DG , Ke YQ , Wilkinson MC and Gallagher JT (1992) Identification of the basic fibroblast growth factor binding sequence in fibroblast heparan sulfate. J Biol Chem 267, 10337–10341. 1587820

[18] Luo YD , Ye S , Kan M and McKeehan WL (2006) Structural specificity in a FGF7‐affinity purified heparin octasaccharide required for formation of a complex with FGF7 and FGFR2IIIb. J Cell Biochem 97, 1241–1258. 16315317 10.1002/jcb.20724

[19] Xu RY , Ori A , Rudd TR , Uniewicz KA , Ahmed YA , Guimond SE , Skidmore MA , Siligardi G , Yates EA and Fernig DG (2012) Diversification of the structural determinants of fibroblast growth factor‐heparin interactions implications for binding specificity. J Biol Chem 287, 40061–40073. 23019343 10.1074/jbc.M112.398826 PMC3501079

[20] Chen G , Gulbranson DR , Yu P , Hou Z and Thomson JA (2012) Thermal stability of fibroblast growth factor protein is a determinant factor in regulating self‐renewal, differentiation, and reprogramming in human pluripotent stem cells. Stem Cells 30, 623–630. 22213113 10.1002/stem.1021 PMC3538808

[21] Zhou C , Guo X , Wang S , Zhu Y and Mu D (2011) Effects of temperature and additives on stability and spectrum of a therapeutic fibroblast growth factor. Daru 19, 138–144. 22615650 PMC3232097

[22] Powell AK , Fernig DG and Turnbull JE (2002) Fibroblast growth factor receptors 1 and 2 interact differently with heparin/heparan sulfate – implications for dynamic assembly of a ternary signaling complex. J Biol Chem 277, 28554–28563. 12034712 10.1074/jbc.M111754200

[23] Vlodavsky I , Folkman J , Sullivan R , Fridman R , Ishaimichaeli R , Sasse J and Klagsbrun M (1987) Endothelial cell‐derived basic fibroblast growth factor: synthesis and deposition into subendothelial extracellular matrix. Proc Natl Acad Sci USA 84, 2292–2296. 3470794 10.1073/pnas.84.8.2292 PMC304636

[24] Battaglia C , Mayer U , Aumailley M and Timpl R (1992) Basement‐membrane heparan sulfate proteoglycan binds to laminin by its heparan sulfate chains and to nidogen by sites in the protein core. Eur J Biochem 208, 359–366. 1521532 10.1111/j.1432-1033.1992.tb17195.x

[25] Sun C , Marcello M , Li Y , Mason D , Levy R and Fernig DG (2016) Selectivity in glycosaminoglycan binding dictates the distribution and diffusion of fibroblast growth factors in the pericellular matrix. Open Biol 6, 150277. 27009190 10.1098/rsob.150277 PMC4821244

[26] Uniewicz KA , Ori A , Xu RY , Ahmed Y , Wilkinson MC , Fernig DG and Yates EA (2010) Differential scanning fluorimetry measurement of protein stability changes upon binding to glycosaminoglycans: a screening test for binding specificity. Anal Chem 82, 3796–3802. 20353159 10.1021/ac100188x

[27] Petitou M , Herault LP , Bernat A , Driguez PA , Duchaussoy P , Lormeau JC and Herbert JM (1999) Synthesis of thrombin‐inhibiting heparin mimetics without side effects. Nature 398, 417–422. 10201371 10.1038/18877

[28] Xu D and Esko JD (2014) Demystifying heparan sulfate‐protein interactions. Annu Rev Biochem 83, 129–157. 24606135 10.1146/annurev-biochem-060713-035314 PMC7851832

[29] Li Y , Sun C , Yates EA , Jiang C , Wilkinson MC and Fernig DG (2016) Heparin binding preference and structures in the fibroblast growth factor family parallel their evolutionary diversification. Open Biol 6, 150275. 27030175 10.1098/rsob.150275 PMC4821243

[30] de Araujo CA , Noseda MD , Cipriani TR , Goncalves AG , Duarte ME and Ducatti DR (2013) Selective sulfation of carrageenans and the influence of sulfate regiochemistry on anticoagulant properties. Carbohyd Polym 91, 483–491. 10.1016/j.carbpol.2012.08.034 23121936

[31] de Raucourt E , Mauray S , Chaubet F , Maiga‐Revel O , Jozefowicz M and Fischer AM (1998) Anticoagulant activity of dextran derivatives. J Biomed Mater Res 41, 49–57. 9641623 10.1002/(sici)1097-4636(199807)41:1<49::aid-jbm6>3.0.co;2-q

[32] Ori A , Free P , Courty J , Wilkinson MC and Fernig DG (2009) Identification of heparin‐binding sites in proteins by selective labeling. Mol Cell Proteomics 8, 2256–2265. 19567366 10.1074/mcp.M900031-MCP200 PMC2758754

[33] Kreuger J , Spillmann D , Li JP and Lindahl U (2006) Interactions between heparan sulfate and proteins: the concept of specificity. J Cell Biol 174, 323–327. 16880267 10.1083/jcb.200604035 PMC2064228

[34] Ori A , Wilkinson MC and Fernig DG (2008) The heparanome and regulation of cell function: structures, functions and challenges. Front Biosci 13, 4309–4338. 18508513 10.2741/3007

[35] Hamer GK and Perlin AS (1976) A 13C‐N.M.R. spectral study of chondroitin sulfates A, B, and C: evidence of heterogeneity. Carbohydr Res 49, 37–48. 963697 10.1016/s0008-6215(00)83123-7

[36] Sun C , Li Y , Taylor SE , Mao X , Wilkinson MC and Fernig DG (2015) HaloTag is an effective expression and solubilisation fusion partner for a range of fibroblast growth factors. PeerJ 3, e1060. 26137434 10.7717/peerj.1060 PMC4485707

[37] Xu RY , Rudd TR , Hughes AJ , Siligardi G , Fernig DG and Yates EA (2013) Analysis of the fibroblast growth factor receptor (FGFR) signalling network with heparin as coreceptor: evidence for the expansion of the core FGFR signalling network. FEBS J 280, 2260–2270. 23441759 10.1111/febs.12201

[38] Sun C , Li Y , Yates EA and Fernig DG (2015) SimpleDSFviewer: a tool to analyse and view differential scanning fluorimetry data for characterising protein thermal stability and interactions. PeerJ PrePrints 3, e1555v1. 10.1002/pro.3703 PMC6933846 31394001

[39] Tian H , Zhao Y , Chen N , Wu M , Gong W , Zheng J , Fernig DG , Jungbauer A , Wang D , Li X *et al* (2016) High production in *E. coli* of biologically active recombinant human fibroblast growth factor 20 and its neuroprotective effects. Appl Microbiol Biotechnol 100, 3023–3034. 26603761 10.1007/s00253-015-7168-y

[40] Mach H , Ryan JA , Burke CJ , Volkin DB and Middaugh CR (1993) Partially structured self‐associating states of acidic fibroblast growth factor. Biochemistry 32, 7703–7711. 7688566 10.1021/bi00081a015

[41] Rahmoune H , Chen HL , Gallagher JT , Rudland PS and Fernig DG (1998) Interaction of heparan sulfate from mammary cells with acidic fibroblast growth factor (FGF) and basic FGF – regulation of the activity of basic FGF by high and low affinity binding sites in heparan sulfate. J Biol Chem 273, 7303–7310. 9516424 10.1074/jbc.273.13.7303

[42] Vlodavsky I , Miao HQ , Medalion B , Danagher P and Ron D (1996) Involvement of heparan sulfate and related molecules in sequestration and growth promoting activity of fibroblast growth factor. Cancer Metastasis Rev 15, 177–186. 8842489 10.1007/BF00437470

